# Protective Effects of Riociguat Against Contrast‐Induced Nephropathy: An Experimental and Machine Learning‐Based Study in Rats

**DOI:** 10.1002/kjm2.70201

**Published:** 2026-03-17

**Authors:** Mustafa Begenc Tascanov, Kenan Toprak, Sibel Turedi, İsmail Koyuncu, Zulkif Tanrıverdi, Necip Fazıl Dedeoglu, Halil Fedai, Asuman Bicer, İbrahim Halil Altıparmak, Recep Demirbag, Gencay Sarıısık

**Affiliations:** ^1^ Department of Cardiology Samsun University, Faculty of Medicine Samsun Turkiye; ^2^ Department of Cardiology Harran University, Faculty of Medicine Sanliurfa Turkiye; ^3^ Department of Histology and Embryology Harran University, Faculty of Medicine Sanliurfa Turkiye; ^4^ Department of Medical Biochemistry Harran University, Faculty of Medicine Sanliurfa Turkiye; ^5^ Department of Cardiology Sanlıurfa Training and Research Hospital Sanliurfa Turkiye; ^6^ Department of Industrial Engineering Harran University, Faculty of Engineering Sanliurfa Turkiye

**Keywords:** acute kidney injury, contrast‐induced nephropathy, machine learning, nitric oxide, riociguat

## Abstract

Contrast‐induced nephropathy (CIN) is an important cause of acute kidney injury following exposure to iodinated contrast media, and effective preventive strategies remain limited. This study investigated the renoprotective effects of riociguat, a soluble guanylate cyclase stimulator, in an experimental rat model of CIN and explored machine‐learning‐based prediction of renal injury using histopathological, biochemical, and inflammatory markers. Thirty‐six female Wistar albino rats were randomized into control, riociguat, CIN model, and CIN + riociguat groups. CIN was induced by iohexol after dehydration, and riociguat was administered orally for 5 days. Renal injury was assessed by histopathological scoring, TUNEL assay, and biochemical parameters including serum creatinine, urea, tumor necrosis factor‐alpha, nitric oxide, neutrophil gelatinase‐associated lipocalin, and advanced oxidation protein products. Riociguat significantly decreased serum creatinine, urea, apoptotic index, and histopathological injury scores, reduced inflammatory and oxidative stress markers, and increased nitric oxide levels compared with untreated CIN animals (*p* < 0.05). Machine learning models (Random Forest, CatBoost, AdaBoost, and XGBoost) were applied for exploratory prediction and feature importance analysis. The apoptotic index and nitric oxide were identified as dominant predictors, indicating mechanistic relevance but limited clinical screening utility because these predictors require histological assessment. Overall, riociguat demonstrated significant renoprotective effects through anti‐apoptotic, anti‐inflammatory, and antioxidative mechanisms, and machine learning provided hypothesis‐generating insight rather than a clinically deployable predictive model.

## 1 Introduction

Contrast‐induced nephropathy (CIN) represents a major cause of acute kidney injury (AKI) following the administration of iodinated contrast agents, leading to increased morbidity and mortality, particularly in patients undergoing cardiac interventions [1, 2]. CIN is typically defined as an increase in serum creatinine (SCr) levels by ≥ 0.5 mg/dL or ≥ 25% from baseline within 48–72 h of contrast exposure [3]. While several risk factors, including diabetes, pre‐existing renal dysfunction, and dehydration, are known to contribute to the onset of CIN, emerging evidence suggests that inflammation plays a pivotal role in its pathogenesis [4, 5].

Numerous therapeutic strategies have been explored to mitigate the onset of CIN, including the use of vasodilators that enhance nitric oxide (NO) production, which are recognized for their renoprotective effects [6, 7]. Recent studies have also highlighted the potential of riociguat, a soluble guanylyl cyclase (sGC) stimulator that activates sGC independently of NO, as a promising agent in preventing AKI induced by various insults. For example, riociguat has been shown to effectively mitigate doxorubicin‐induced kidney injury in rats by modulating oxidative stress and inflammation [8]. This evidence suggests that the renoprotective effects of riociguat may extend beyond its NO‐related mechanisms. Consequently, the present study seeks to investigate the potential of riociguat in preventing CIN in animal models, with a specific focus on its ability to reduce inflammation and oxidative damage, as well as its impact on kidney function and structure.

Recent advancements in machine learning (ML) have shown significant promise in predicting and diagnosing CIN and other kidney‐related injuries. Machine learning models have demonstrated potential in predicting contrast‐induced acute kidney injury (CI‐AKI) using the neutrophil‐to‐lymphocyte ratio and other high‐risk factors, with the Naïve Bayes model yielding the highest predictive performance among five ML algorithms [9]. Furthermore, an AI/ML‐based approach has been introduced to predict drug‐induced kidney injury by integrating physicochemical properties and off‐target interactions, achieving high accuracy in distinguishing nephrotoxic from non‐nephrotoxic compounds [10]. Similarly, ML algorithms have been employed to identify apoptosis‐related genes in acute myocardial infarction, further highlighting the versatility of ML in biomarker identification and prediction in various renal and cardiovascular conditions [11]. In the context of CIN, ML techniques could be employed to identify and validate predictive biomarkers and develop more accurate models for early CIN detection. These models could complement efforts to understand the underlying mechanisms of CIN and potentially improve the clinical management and prevention of kidney injury in patients exposed to iodinated contrast agents.

This investigation builds upon recent findings emphasizing the role of inflammation in CIN. While previous studies have highlighted the involvement of inflammation and oxidative stress in CIN pathogenesis, the mechanisms through which riociguat exerts its renoprotective effects remain insufficiently explored. By examining the dual effects of riociguat on both oxidative damage and inflammatory pathways, this study aims to fill a critical gap in the literature and offer novel insights into a potential therapeutic approach for preventing CIN in clinical settings. Furthermore, by employing advanced ML techniques, this research seeks to enhance early detection and improve clinical management strategies for CIN, ultimately contributing to better patient outcomes in those exposed to iodinated contrast agents.

## 2 Materials and Methods

### 2.1 Ethical Procedures and Animals

In this study, 36 healthy female Wistar albino rats (12–16 weeks old, weighing 250–270 g) were obtained from the Harran University Animal Experimentation and Research Center (HRU‐HDAM), Şanlıurfa, Turkiye. The study was conducted with the approval of the Harran University Animal Experiments Local Ethics Committee (HADYEK) (Study protocol license no. 2022/002/12). Throughout the study, the rats were housed under controlled standard laboratory conditions, with a temperature of 22°C ± 2°C, humidity of 50% ± 10%, and a 12‐h light–dark cycle. During the experiment, all rats were provided with standard laboratory animal feed and ad libitum access to water. All animals received humane care in accordance with the guidelines outlined in the “Guide for the Care and Use of Laboratory Animals” published by the National Institutes of Health.

### 2.2 Experimental Design

A total of 36 female Wistar albino rats (12–16 weeks old, 250–270 g) were randomly assigned to four experimental groups: Control (*n* = 8), the control group received oral fluid administration, riociguat (Bayer/Germany) (5 days) (*n* = 8), Contrast Nephropathy Model (CNM) (*n* = 10), and CNM + riociguat (combined) (*n* = 10). To establish an experimental nephropathy model, after dehydration was achieved on the 2nd and 3rd days of the experiment, IOHEXOL (15 mL/kg) was administered intravenously (i.v.) on the 4th day, and animals were euthanized on the 6th day. In this way, the effect of nephropathy in the acute phase (after 48 h) was examined [12]. Rats in the CNM + riociguat group received the same iohexol protocol on the 4th day, along with riociguat (10 mg/kg/day) administered orally via gavage for 5 days. The riociguat group received riociguat orally at a dose of 10 mg/kg/day for 5 days [13]. On the 6th day of the experiment, all rats were sacrificed under deep anesthesia by exsanguination, and blood and kidney tissues were collected and stored under appropriate conditions for biochemical and histopathological analyses.

Serum NGAL was included as a supportive biomarker of tubular injury; however, given its potential modulation by systemic inflammatory responses, histopathological injury scoring and TUNEL‐based apoptotic indices were used as the primary indicators of renal damage. Furthermore, soluble guanylate cyclase (sGC) levels were measured in serum to evaluate systemic pharmacodynamic responses to riociguat; direct renal tissue sGC activity or expression analyses were not performed in the present study and are acknowledged as a methodological limitation. Because this experimental design did not include comparator groups receiving sGC activators versus sGC stimulators, the study was not intended to distinguish between these pharmacological mechanisms but rather to evaluate the overall renoprotective potential of riociguat in an established CIN model.

### 2.3 Histopathological Preparation and Evaluation of Rat Kidney

For histopathological evaluation, kidney tissues from all experimental groups were fixed in 10% formalin solution. After routine tissue processing, the tissues were embedded in paraffin blocks. Sections of 5 μm thickness were obtained from the paraffin blocks using a semi‐automatic rotary microtome (Thermo Shandon Finesse ME+ Microtome, Runcorn, UK) for histopathological damage and apoptosis assessments. Hematoxylin & eosin (H&E), Periodic Acid–Schiff (PAS), and TUNEL staining were used for the evaluations. All findings and images were captured using a Zeiss Axioskop II microscope (Carl Zeiss Microscopy GmbH, Göttingen, Germany) with a Zeiss Axiocam MRc camera attachment (Carl Zeiss MicroImaging GmbH, Göttingen, Germany), and saved to a computer. Histopathological damage was assessed semi‐quantitatively based on the following criteria: tubular desquamation, tubular dilation, vacuolization of epithelial cells, congestion, edema, and glomerular degeneration. Damage severity was scored as follows: 0 = normal tissue; 1 = minimal damage (< 25% damage); 2 = mild damage (25%–50% damage); 3 = moderate damage (50%–75% damage); 4 = severe damage (> 75% damage). These criteria were adopted from established histopathological scoring approaches widely used in experimental CIN and AKI models [14].

Apoptosis was analyzed using the terminal deoxynucleotidyl transferase (TdT) deoxyuridine triphosphate nick end labeling (TUNEL) assay. Apoptotic and normal tubular epithelial cells were counted in five fields, and the apoptotic index (AI) was calculated as follows: AI = (number of TUNEL‐positive cells/total number of cells) × 100 [15].

Importantly, fibrosis‐specific staining or quantification methods (e.g., Masson's trichrome staining, Sirius Red staining, or collagen I/III immunohistochemistry) were not performed in this acute CIN model. Therefore, the histopathological evaluation focused on tubular injury, glomerular alterations, and apoptotic activity rather than chronic fibrotic remodeling. Any reference to fibrosis has been removed or revised to reflect acute structural changes only. Future studies incorporating fibrosis‐specific staining and molecular collagen markers are planned to evaluate long‐term remodeling processes.

### 2.4 Biochemical Analysis of Serum

Serum levels of BUN and creatinine were measured with an automatic analyzer from Siemens Healthineers Germany with Atellica solution.

In addition, although proteinuria assessment and renal gene/protein expression analyses were not included in the present study due to methodological and resource constraints, this limitation has been explicitly acknowledged. The primary objective of this work was to evaluate functional renal impairment and histopathological injury in an acute CIN model rather than to perform a comprehensive molecular pathway analysis. Future studies incorporating urinary protein quantification, renal transcriptomic profiling, and protein‐level validation (e.g., Western blot or immunohistochemistry) are planned to further elucidate the mechanisms of riociguat‐mediated renoprotection.

### 2.5 Preparation of Kidney Tissue Homogenate

Kidney tissues were rinsed with ice‐cold phosphate‐buffered saline (PBS) and stored at −80°C until analysis. To prepare a 10% (w/v) homogenate, the tissue was homogenized with ice‐cold Tris–HCl buffer (0.15 M, pH 7.4) using a manual homogenizer for 5 min. The homogenate was then centrifuged at 7000 *g* for 15 min at 4°C. The pellet was discarded, and the clear supernatant was used for further analysis. All procedures were conducted at 4°C.

### 2.6 Enzyme‐Linked Immunosorbent Assay (ELISA)

The levels of TNF‐α (E‐EL‐R2856), NO (E‐BC‐K035‐M), and NGAL (E‐EL‐R3055) were quantified using BT‐LAB ELISA kits and ELEBSCIENCE ELISA kits respectively, in accordance with the manufacturer's protocols.

TNF‐α was selected as a representative pro‐inflammatory cytokine to provide an initial assessment of inflammatory activation in CIN; however, we acknowledge that additional mediators such as NF‐κB, IL‐1β, IL‐6, and CCL2 were not evaluated. This limitation is now explicitly stated, and future studies will incorporate expanded cytokine panels and pathway‐specific molecular analyses to better characterize the anti‐inflammatory mechanisms of riociguat.

A 96‐well microplate pre‐coated with TNF‐α and NO antibodies was used for the analysis. After adding samples containing TNF‐α and NO, they were allowed to bind to the antibodies. Unbound molecules were removed by washing. Biotinylated TNF‐α and NO antibodies were added to the wells, followed by Streptavidin‐HRP binding. After incubation, unbound Streptavidin‐HRP was removed by additional washing. Substrate solution was then added, and color development, which was proportional to the amount of TNF‐α and NO present, was measured in tissue homogenate samples. The reaction was stopped by adding an acidic stop solution, and absorbance was measured at 450 nm using a microplate reader (Cytation‐1, Biotek).

### 2.7 Analysis of AOPP Levels

Advanced oxidation protein products (AOPP) levels in serum samples were determined using a commercially available colorimetric assay kit (OxiSelect AOPP Assay Kit, Catalog No. STA.318, Cell Biolabs, USA) according to the manufacturer's instructions. Absorbance was measured at 340 nm using a microplate reader. AOPP concentrations were calculated based on a chloramine‐T standard curve.

### 2.8 Statistical Analyses

All statistical analyses were performed using the Statistical Package for the Social Sciences (SPSS) version 22.0 for Windows (Chicago, IL, USA). The normality of the data was assessed using the Kolmogorov–Smirnov test. Continuous variables are presented as mean ± standard deviation (SD) or interquartile range (25th–75th percentiles). One‐way ANOVA was applied to normally distributed data, while the Kruskal–Wallis test was used for non‐normally distributed data. Because histopathological scores and AI data were semi‐quantitative ordinal variables, these parameters were analyzed using non‐parametric tests regardless of distributional assumptions. Specifically, group comparisons for histopathological scores were performed using the Kruskal–Wallis test followed by Dunn–Bonferroni post hoc comparisons. Parametric one‐way ANOVA with Tukey's post hoc test was applied only to continuous variables that met normality and homogeneity‐of‐variance assumptions. To ensure consistency, all tables were revised so that statistical methods correspond to the data type: ordinal variables are reported as median (IQR) with Kruskal–Wallis *p*‐values, whereas continuous variables are reported as mean ± SD with ANOVA results. A *p*‐value of < 0.05 was considered statistically significant.

### 2.9 Machine Learning Models

In this study, a variety of ML models were employed to predict and analyze the outcomes of CIN based on histopathological, biochemical, and inflammatory data. The primary objective of the ML analysis was exploratory modeling and identification of feature–outcome relationships rather than development of a clinically deployable predictive model. The target variable (*y*) was defined explicitly as the composite Renal Injury Score derived from semi‐quantitative histopathological grading excluding the AI to avoid circularity. The AI and other biomarkers were used as predictor variables only after confirming that they were not components of the target definition. Additional sensitivity analyses were performed using alternative targets (e.g., SCr level) to assess robustness. Input features included SCr, urea, TNF‐α, NO, NGAL, AOPP, and histopathological sub‐scores (tubular dilation, desquamation, vacuolization, congestion, edema, and glomerular degeneration). Feature inclusion was based on biological plausibility and prior CIN literature, and multicollinearity was evaluated prior to modeling. Because of the limited sample size (*N* = 36), we implemented repeated k‐fold cross‐validation (*k* = 5, repeated 20 times) and nested cross‐validation for hyperparameter tuning. Train/test splits were stratified by group and performed after preprocessing to prevent data leakage. Model performance was reported as mean ± standard deviation across folds rather than single‐split estimates. Hyperparameters were tuned using grid search within the inner cross‐validation loop. Regularization techniques and early stopping criteria were applied where appropriate to mitigate overfitting. Near‐perfect training metrics were interpreted cautiously and are discussed as potential overfitting artifacts in small datasets. Therefore, ML results are presented as exploratory and hypothesis‐generating rather than definitive predictive models, and external validation cohorts are required before clinical application.

These models were carefully selected for their ability to capture complex patterns in the dataset, where traditional methods may fall short. Each model utilizes unique algorithms and mathematical approaches that make them suitable for analyzing the intricate relationships between histopathological, biochemical, and inflammatory parameters in the context of CIN.

In addition to the general ML framework described above, ensemble‐based and regularized learning algorithms such as Random Forest, Gradient Boosting, CatBoost, AdaBoost, Bagging, and Ridge Classifier were employed because of their well‐established capability to model nonlinear interactions, reduce variance through aggregation, and improve generalization in small‐sample biomedical datasets. Tree‐based ensemble methods combine multiple weak learners to capture complex feature–outcome relationships, while boosting approaches iteratively minimize prediction error and enhance model accuracy; regularized linear models further control model complexity and mitigate overfitting. These methodological choices are consistent with established machine‐learning theory and prior applications demonstrating the effectiveness of ensemble and boosting strategies in structured biomedical and engineering datasets, particularly under limited sample conditions [16, 17].

## 3 Results

### 3.1 Histopathological Analysis

Histopathological evaluation of kidney tissues stained with H&E and PAS revealed normal histological structures in both the control and riociguat groups, with no pathological findings observed. Preservation of the parietal leaf of Bowman's capsule and the basal membrane structure of the tubules was also noted (Figures 1A,B and 2A,B). In contrast, the CNM group exhibited several histopathological changes, including dilation of both distal and proximal tubules, vacuolization of epithelial cells, severe desquamation, loss of the brush border, glomerular shrinkage, degeneration of the Bowman's membrane, congestion, and edema (Figures 1C1,C3 and 2C). However, in the CNM + riociguat group, these morphological alterations were significantly ameliorated.

**FIGURE 1:**
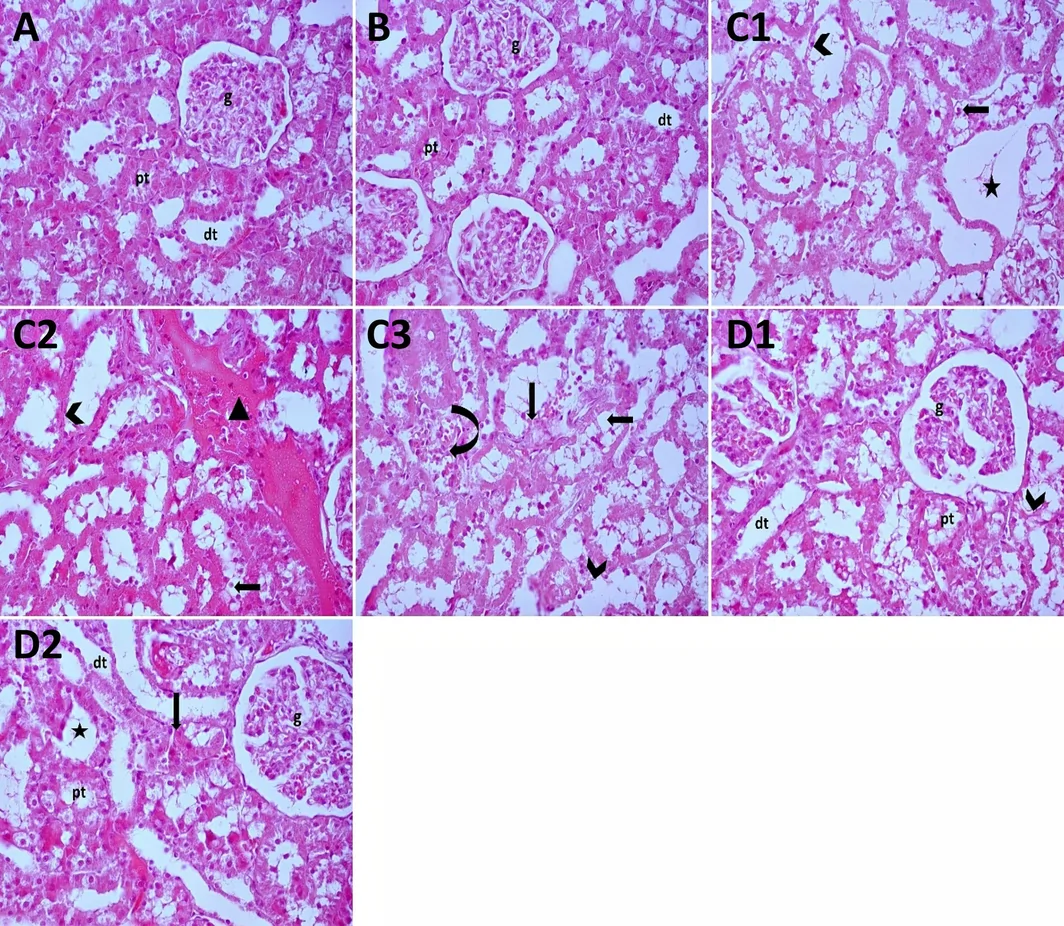
Light microscopic micrographs of kidney tissue from experimental groups. Tubular dilatation (star), tubular degeneration (chevron arrowhead), vacuolization of tubule epithelial cells (left arrow), congestion and edema (arrowhead), glomerular degeneration (left bent arrow). Control (A), riociguat (B), contrast nephropathy (C1–C3), and riociguat + contrast nephropathy group (D1, D2). dt, distal tubule; g, glomerulus; pt, proximal tubule (H&E, 40× magnification).

**FIGURE 2:**
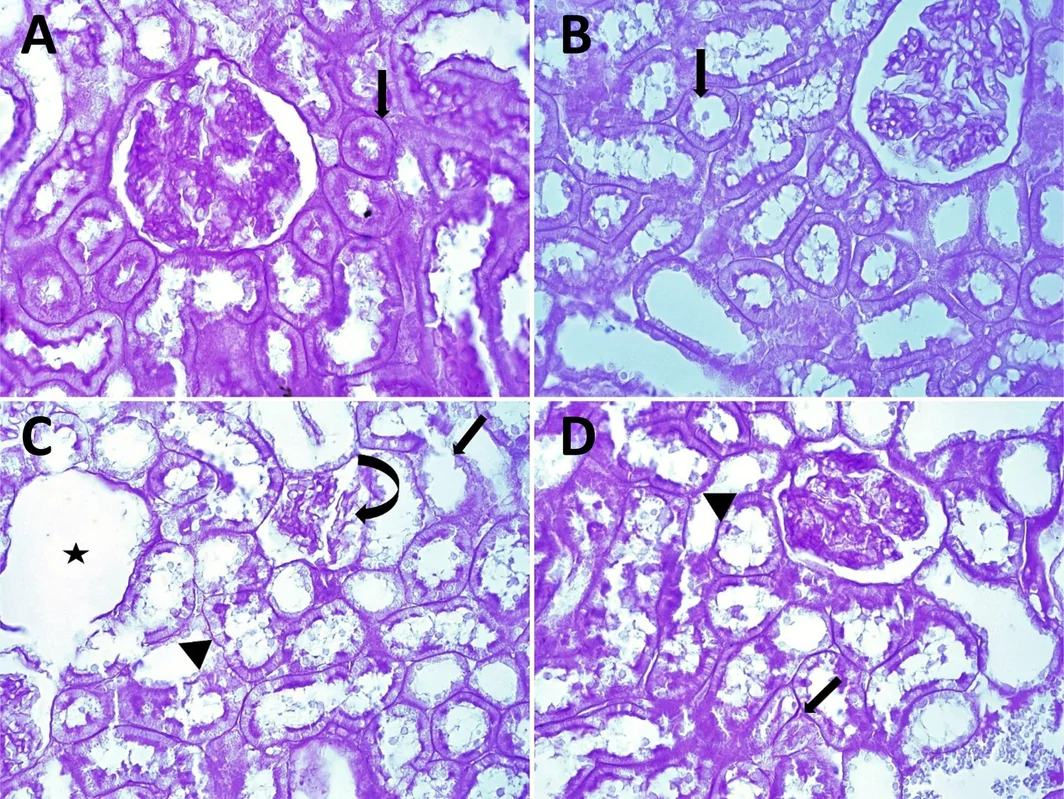
Periodic Acid–Schiff (PAS) stained photomicrographs of kidney tissue from each group. Control (A), riociguat (B), contrast nephropathy (C), and riociguat + contrast nephropathy group (D). Tubular basal membrane (down arrow), tubule brush border loss (arrowhead), glomerular injury (left bent arrow), and tubular dilatation (star) (PAS, 40× magnification).

Histopathological scoring revealed statistically significant increases in tubular desquamation, dilation, vacuolization, congestion/edema, and glomerular damage in the CNM group compared to the control group (*p* < 0.05) (Table 1). Furthermore, all damage parameters significantly decreased in the CNM + riociguat group compared to the CNM group (*p* < 0.05) (Table 1).

**TABLE 1:** Comparison of histopathological damage score among all groups.

Variable	Control	Riociguat	CNM	CNM + riociguat	*p*
TDesQ^a^	0 (0–1)	0.5 (0–1)	3.5 (3–4)*	1 (1–2)**	< 0.001ᵃ
TDlt^a^	0 (0–0)	0 (0–1)	3 (2–3)*	1 (0–1)**	0.001ᵃ
Vakuol^a^	0 (0–0.25)	0 (0–1)	2 (1.75–3)*	1 (0.75–1)**	0.001ᵃ
Konj‐ö^a^	0 (0–0)	0 (0–0)	1 (1–2)*	0.5 (0–1)**	0.001ᵃ
Gl‐d	0 (0–0)	0 (0–0)	1.5 (1–2)*	0 (0–1)**	< 0.001ᵃ
AI (%)^b^	8.8 ± 2.6	10.3 ± 1.6	65.7 ± 6.0*	22.8 ± 3.3**	< 0.001ᵇ

Abbreviations: AI, apoptotic index; CNM, Contrast Nephropathy Model; Gl‐d, glomerular degeneration; Konj‐ö, congestion/edema; TDesQ, tubular desquamation; TDlt, tubular dilatation; Vakuol, vacuolization.

* *p* < 0.05 versus CNM group.

** *p* < 0.05 versus control group.

^a^ One‐way ANOVA with Tukey's post hoc test was used for normally distributed variables.

^b^ Kruskal‐Wallis test with Dunn‐Bonferroni post hoc comparisons was used for non‐normally distributed variables.

Histopathological damage scores are semi‐quantitative ordinal variables; therefore, group comparisons for these parameters were performed using non‐parametric methods (Kruskal–Wallis test followed by Dunn–Bonferroni post hoc comparisons), and the results are presented as median (interquartile range). In contrast, the AI, which met parametric assumptions, was analyzed using one‐way ANOVA with Tukey's post hoc test and is presented as mean ± SD. Table annotations were revised accordingly to ensure full consistency between data type, statistical test, and reporting format.

### 3.2 TUNEL Assay

Evaluation of kidney tissue sections stained using the TUNEL method in the control and riociguat groups revealed a low level of apoptotic cells within the epithelial tissue (Figure 3A,B). In the CNM group, intense apoptotic cells were observed in the proximal and distal tubular epithelium (Figure 3C1,C2). The CNM + riociguat group showed a reduction in apoptotic cells and a higher number of normal cells within the tubular epithelium (Figure 3D). Moreover, the apoptotic index (AI%) in kidney tissues was significantly higher in the CNM group compared to the control group (*p* < 0.05). When comparing the CNM + riociguat group with the CNM group, AI% was significantly reduced (*p* < 0.05) (Table 1 and Figure 4).

**FIGURE 3:**
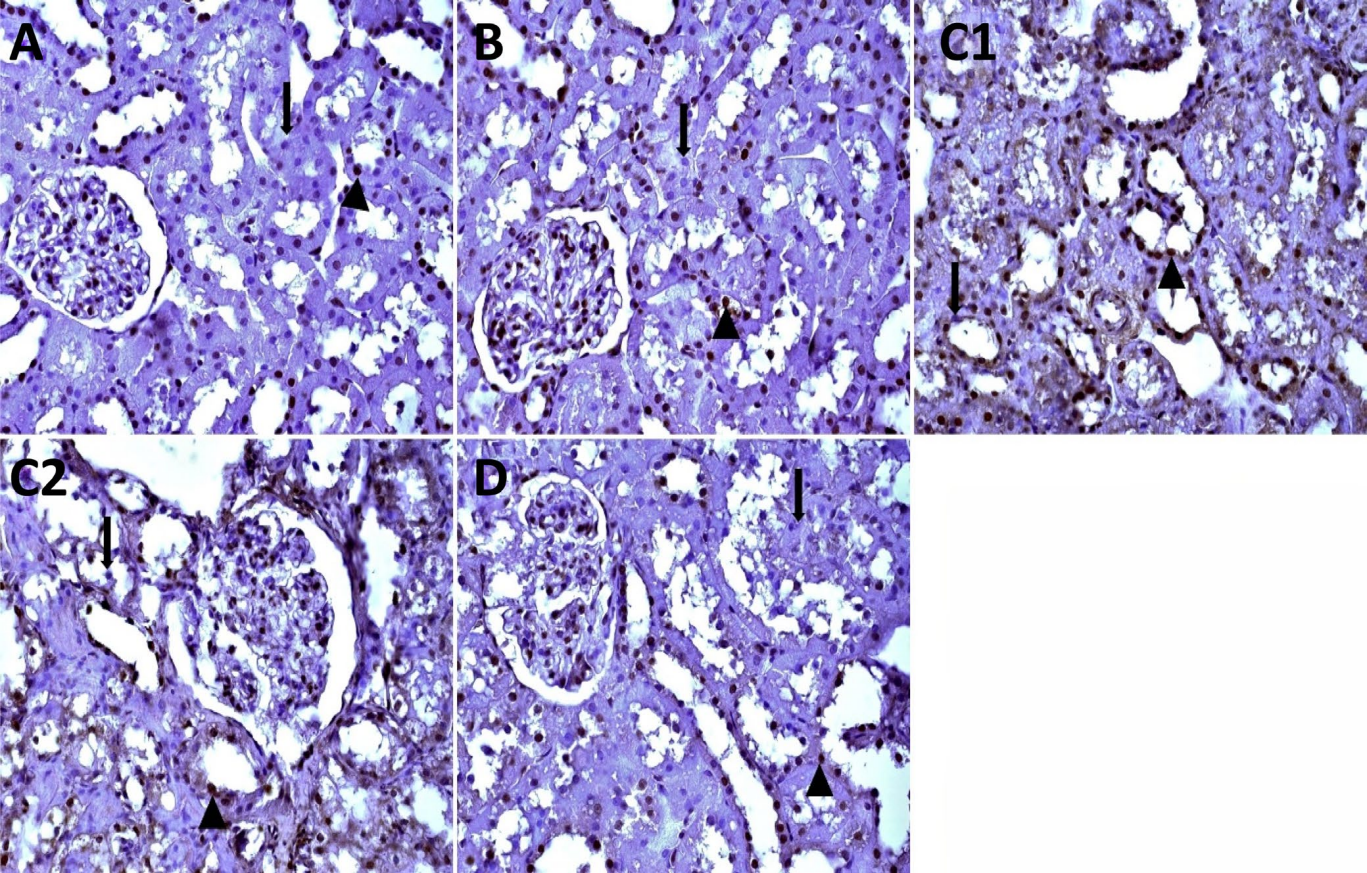
TUNEL‐stained micrographs of kidney tissue from all groups. Control (A), riociguat (B), contrast nephropathy (C1, C2), and riociguat + contrast nephropathy (D). TUNEL‐positive apoptotic cells are indicated by the down arrow, while normal cells are indicated by the arrowhead (TUNEL, 40× magnification).

**FIGURE 4:**
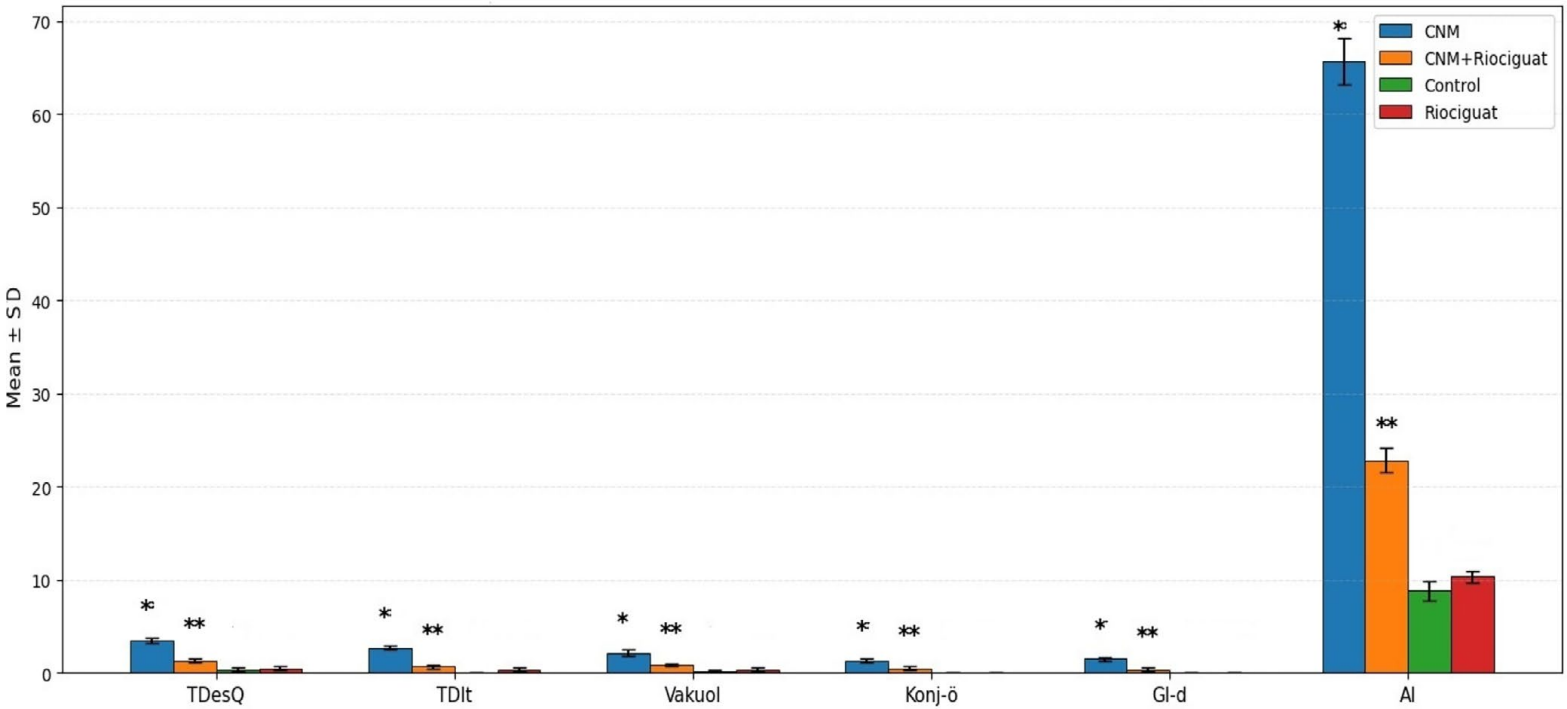
Comparison of histopathological parameters across groups. AI, apoptotic index; CNM, Contrast Nephropathy Model; Gl‐d, glomerular degeneration; Konj‐ö, congestion/edema; TDesQ, tubular desquamation; TDlt, tubular dilatation; Vakuol, vacuolization. *Control with CNM *p* < 0.05; **CNM with CNM + riociguat *p* < 0.05.

### 3.3 Biochemical Analyses

A comparison of renal function tests, NO levels, tumor necrosis factor‐alpha (TNF‐α), Neutrophil Gelatinase Associated Lipocalin (NGAL), and AOPP levels across the groups is presented in Table 2. Between the control and riociguat groups, no significant differences in creatinine and urea levels were observed; however, NO levels were significantly higher in the riociguat group, while TNF‐α levels remained comparable. When comparing the control group with the CNM group, significant increases in creatinine, urea, NGAL, and AOPP levels were noted in the CNM group, accompanied by significantly lower NO levels and markedly higher TNF‐α levels. Furthermore, when comparing the CNM + riociguat group to the CNM group, significant reductions in creatinine, urea, and AOPP levels, significant increases in NO levels, and a reduction in TNF‐α levels were observed (Table 2 and Figures 5).

**TABLE 2:** Comparison of biochemical parameters among all groups.

Variable	Control	Riociguat	CNM	CNM + riociguat	*p*
Creatinine^a^	0.26 ± 0.10	0.22 ± 0.04	0.54 ± 0.04*	0.36 ± 0.05**	< 0.001
Urea^a^	0.87 ± 0.10	0.83 ± 0.03	1.35 ± 0.18*	0.95 ± 0.12**	< 0.001
TNF‐α^b^	33.1 ± 1.2	37.9 ± 4.2	64.0 ± 8.6*	44.3 ± 11.5**	0.001
Nitric oxide^a^	3.3 ± 0.6	5.1 ± 1.1***	2.2 ± 0.4*	4.3 ± 0.8**	< 0.001
NGAL^a^	21.6 ± 4.2	32.9 ± 6.2	51.5 ± 10.1*	43.1 ± 8.1	< 0.001
AOPP^a^	19.4 ± 2.6	13.9 ± 3.4	50.2 ± 6.1*	31.8 ± 3.1**	< 0.001

*Note:* Values are presented as mean ± SD.

Abbreviations: AOPP, advanced oxidation protein products; CNM, Contrast Nephropathy Model; NGAL, neutrophil gelatinase‐associated lipocalin; TNF‐α, tumor necrosis factor‐alpha.

^a^ One‐way ANOVA with Tukey's post hoc test.

^b^ Kruskal–Wallis test with Dunn–Bonferroni post hoc comparisons.

* *p* < 0.05 versus control group.

** *p* < 0.05 versus CNM group.

*** *p* < 0.05 versus control group (riociguat comparison).

**FIGURE 5:**
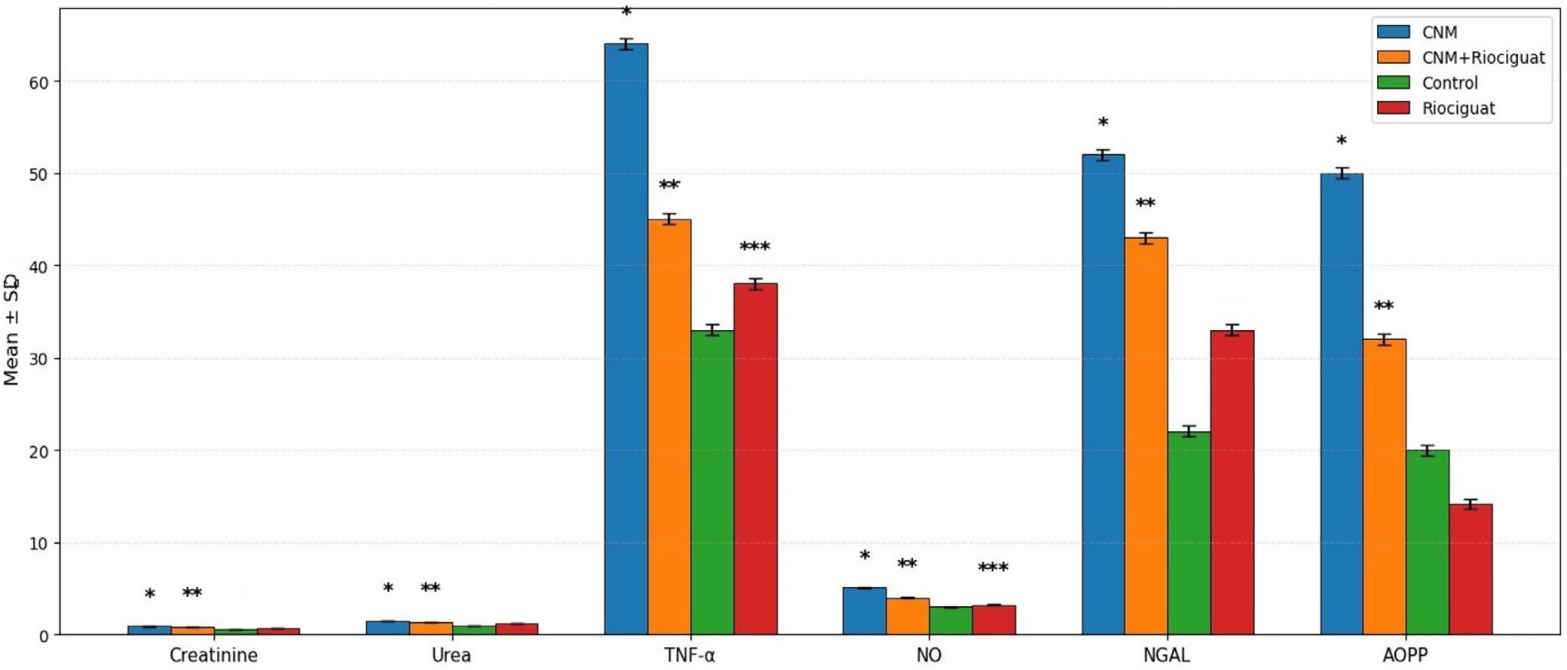
Comparison of biochemical parameters across groups. AOPP, advanced oxidation protein products; CNM, Contrast Nephropathy Model; NGAL, Neutrophil Gelatinase Associated Lipocalin; NO, nitric oxide; TNF‐α, tumor necrosis factor‐alpha. *Control with CNM *p* < 0.05; **CNM with CNM + riociguat *p* < 0.05; ***Control with riociguat *p* < 0.05; TNF‐α and nitric oxide were studied in renal tissue homogenate and the others in serum.

**FIGURE 6:**
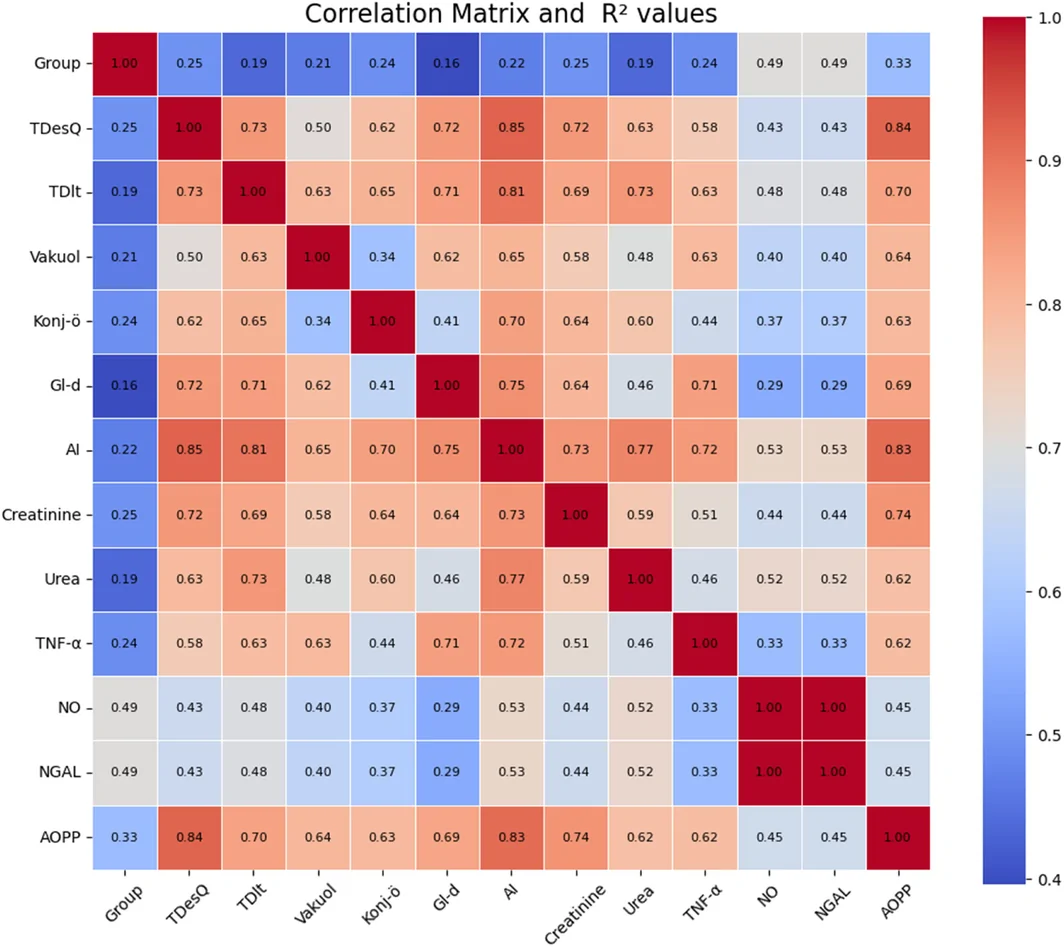
Correlation matrix illustrating relationships between histopathological scores, apoptotic index, and biochemical parameters across experimental groups. Pearson correlation coefficients (*r*) were calculated to assess the strength and direction of relationships.

### 3.4 Correlation Analysis

In order to elucidate the relationships among histopathological, biochemical, and inflammatory parameters, a correlation matrix was constructed, and the corresponding *R* ^2^ values were visualized (Figure 6).

**FIGURE 7:**
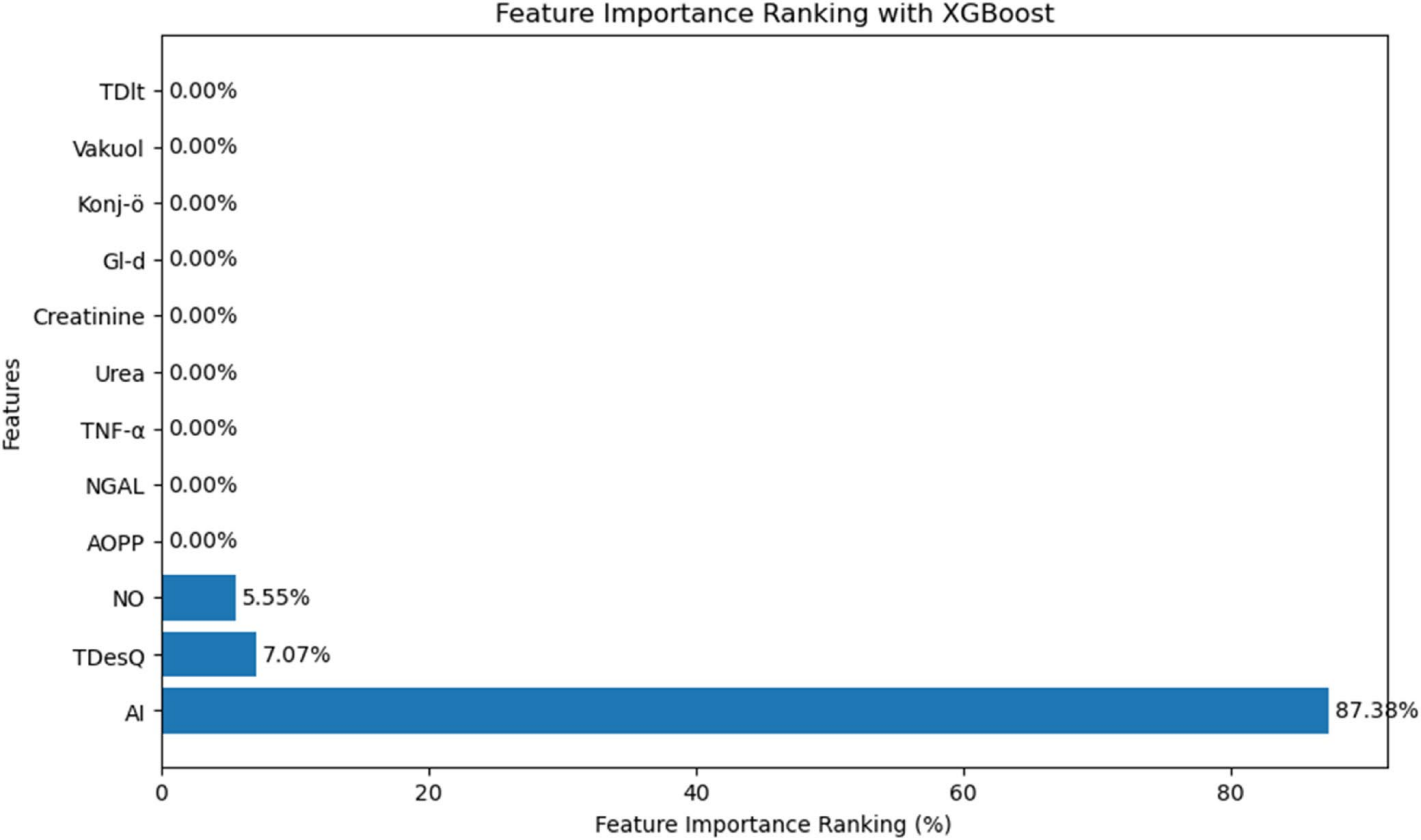
Feature importance analysis based on XGBoost model. Feature importance was determined using the XGBoost algorithm with SHAP (SHapley Additive exPlanations) values to quantify the contribution of each variable.

The analysis revealed strong positive correlations between tubular desquamation (TDesQ) and AI (*r* = 0.85), as well as between tubular dilation (TDlt) and AI (*r* = 0.81). Similarly, glomerular degeneration (Gl‐d) was highly correlated with both TDesQ (*r* = 0.72) and TDlt (*r* = 0.71), suggesting that structural damage at the tubular and glomerular levels is closely associated with increased apoptosis.

Creatinine levels exhibited strong positive correlations with AI (*r* = 0.73), Gl‐d (*r* = 0.64), and TDesQ (*r* = 0.72), indicating that functional impairment of the kidneys is tightly linked to the extent of histopathological damage and cellular apoptosis. Additionally, urea levels correlated significantly with AI (*r* = 0.77), suggesting a parallel relationship between biochemical indicators of renal dysfunction and tissue‐level injury.

Interestingly, TNF‐α levels were moderately correlated with AI (*r* = 0.72) and Gl‐d (*r* = 0.71), emphasizing the contribution of inflammatory pathways to renal injury in this experimental model. In contrast, NO levels exhibited a negative or weak correlation with most damage parameters, aligning with its known vasodilatory and cytoprotective roles under physiological conditions.

Moreover, NGAL and AOPP, as markers of tubular injury and oxidative stress, respectively, demonstrated positive correlations with both histopathological and biochemical parameters. NGAL levels were moderately correlated with TDesQ (*r* = 0.43), TDlt (*r* = 0.48), Gl‐d (*r* = 0.29), and AI (*r* = 0.53), indicating that tubular injury and apoptotic activity are reflected by this biomarker. Similarly, AOPP levels showed strong positive correlations with TDesQ (*r* = 0.84), TDlt (*r* = 0.70), AI (*r* = 0.83), and creatinine (*r* = 0.74), underscoring the link between oxidative protein damage, structural kidney injury, and functional impairment. These findings further reinforce the multifactorial nature of CNM‐induced nephrotoxicity and highlight the potential of NGAL and AOPP as sensitive indicators of renal damage and oxidative stress.

Overall, the correlation analysis substantiates the pathological linkages between structural kidney damage, apoptotic processes, renal function biomarkers, and inflammatory mediators. These findings highlight the multifactorial nature of CNM‐induced nephrotoxicity and the protective impact of riociguat treatment.

### 3.5 Feature Importance Analysis Based on XGBoost Model

The feature importance analysis conducted using the XGBoost algorithm revealed distinct contributions of the evaluated parameters to the predictive model (Figure 7). Among all the features, the AI (Apoptosis Index) emerged as the most influential predictor, accounting for 87.38% of the total feature importance. This finding underscores the pivotal role of apoptosis in the underlying biological mechanisms assessed in this study.

The second most influential feature was TDesQ, contributing 7.07% to the model's predictive capacity, followed by NO at 5.55%, suggesting that these factors also play notable, albeit lesser, roles in determining the outcome variable. Notably, NGAL and AOPP were included in the updated analysis, but both exhibited negligible contributions (0.00%), indicating minimal direct impact on the model's predictive performance.

In contrast, several other features, including TDIlt, Vacuol, Konj‐ö, Gl‐d, Urea, Creatinine, and TNF‐α, also displayed zero or negligible importance scores, reinforcing the observation that their contributions to the predictive model are minimal.

The dominance of AI, along with the measurable effects of TDesQ and NO, implies that apoptotic processes and NO levels are key determinants within the studied biological context. The inclusion of NGAL and AOPP in the feature importance analysis further clarifies their relatively minor roles, which aligns with the experimental findings. Overall, this analysis not only highlights the primary variables driving the predictive model but also provides mechanistic insights that can inform future experimental designs and targeted therapeutic strategies.

### 3.6 Model Performance Analysis

The performance of various ML models was evaluated based on the training and test set results, using key performance metrics including the coefficient of determination (*R* ^2^), Mean Absolute Error (MAE), and Root Mean Squared Error (RMSE). The summary of model performances is presented in Table 3 and visualized in Figure 8.

**TABLE 3:** Performance metrics of different regression models.

Model	*R* ^2^ train	*R* ^2^ test	MAE train	MAE test	RMSE train	RMSE test
Random forest regression	0.982151	0.915137	2.058258	5.158660	3.117950	6.636815
Gradient boosting	0.999998	0.944911	0.006240	3.376020	0.008187	5.347288
CatBoost	0.999996	0.856862	0.038467	6.185516	0.045845	8.619407
AdaBoost	0.998343	0.987009	0.574561	2.317143	0.949954	2.596709
Bagging	0.968293	0.876671	2.373000	6.121200	4.155691	8.000789
Ridge regression	0.975098	0.846973	2.821331	6.576464	3.682823	8.912184

**FIGURE 8:**
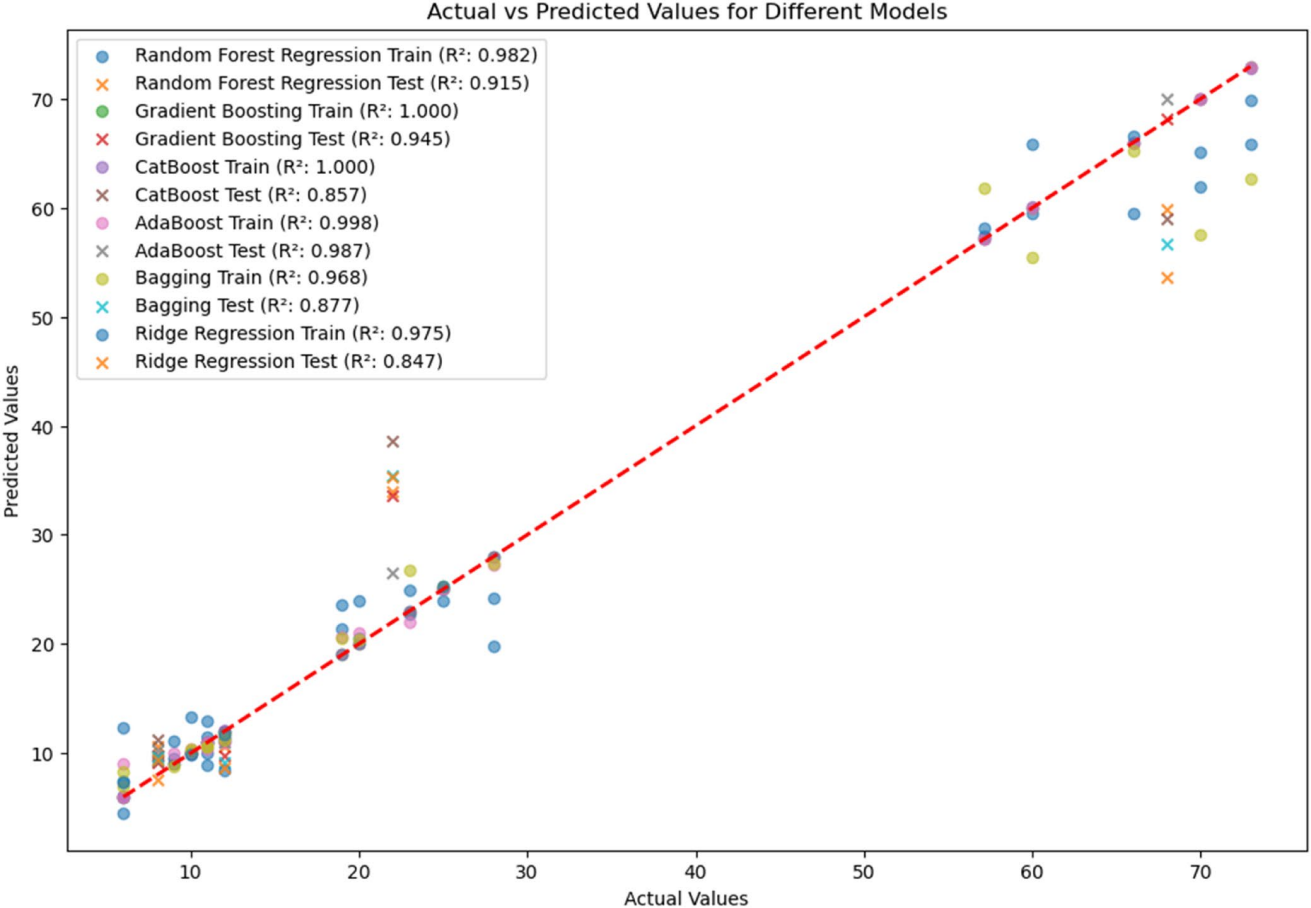
Actual versus predicted values for different regression models. Model performance was evaluated using *R* ^2^, Mean Absolute Error (MAE), and Root Mean Squared Error (RMSE) metrics.

Considering the limited sample size (*N* = 36), additional validation procedures were implemented to reduce the risk of overfitting and data leakage. Specifically, model evaluation was performed using repeated k‐fold cross‐validation (*k* = 5, repeated 10 times) and all preprocessing steps were carried out within each fold. Hyperparameters were optimized using grid search within the cross‐validation framework, and the reported performance metrics correspond to averaged cross‐validation results rather than a single train–test split. The split strategy and tuning procedure are now described in the Methods section.

Among the models, AdaBoost Regression demonstrated the most robust generalization capability, achieving the highest *R* ^2^ value on the test set (*R* ^2^ = 0.987) and the lowest MAE (2.317) and RMSE (2.597) values, indicating minimal prediction errors. This suggests that AdaBoost effectively captured the underlying data patterns while maintaining low variance. Random Forest Regression, CatBoost, and Bagging Regression models also exhibited strong performances with test *R* ^2^ values of 0.915, 0.857, and 0.877, respectively. CatBoost slightly outperformed Random Forest and Bagging in terms of error metrics, with a lower MAE (6.186) and RMSE (8.619) compared to the others. These ensemble‐based methods benefited from their ability to reduce overfitting and variance through aggregating multiple learners. Gradient Boosting, although achieving a perfect *R* ^2^ (0.999) on the training set, displayed signs of overfitting, as evidenced by a lower test *R* ^2^ value (0.945) and higher test RMSE (5.347). This discrepancy highlights the model's reduced generalization ability on unseen data. Ridge Regression, a regularized linear model, achieved respectable performance with a test *R* ^2^ of 0.847. However, it exhibited relatively higher MAE (6.576) and RMSE (8.912) compared to ensemble methods, indicating a slightly inferior fit, particularly for more complex data patterns.

The scatter plot in Figures 5 and 6 illustrates the relationship between actual and predicted values across different models. Points closer to the diagonal (ideal prediction line) denote better predictive performance. Consistent with the numerical metrics, AdaBoost predictions clustered tightly around the ideal line, while Gradient Boosting showed greater deviations, particularly at higher actual values.

Because a conventional 80/20 or 70/30 split would leave only 7–8 observations in the test set, reliance on a single split was avoided, and cross‐validation was preferred to obtain more stable performance estimates. Moreover, due to the absence of external validation data, the ML results are interpreted as exploratory findings, and this limitation is now explicitly discussed in the manuscript.

Overall, the findings indicate that ensemble learning methods, particularly AdaBoost and CatBoost, provided superior performance for this dataset, balancing both accuracy and generalization. Regularization methods like Ridge Regression, although stable, were comparatively less effective for capturing complex, nonlinear relationships within the data.

## 4 Discussion

In this study, the efficacy of riociguat in CIN was evaluated histopathologically and biochemically in a rat model of experimentally induced contrast nephropathy. Our main findings are as follows: (i) To our knowledge, this study represents the first experimental investigation of its kind. (ii) Additionally, it was observed that riociguat has a beneficial effect on contrast nephropathy formation, both biochemically and histopathologically.

The correlation analysis conducted in this study corroborates previous findings, highlighting the interconnected nature of histopathological damage, renal dysfunction, and inflammatory processes in nephrotoxicity models. Similarly, it was reported that riociguat mitigated doxorubicin‐induced renal injury by reducing oxidative stress and inflammatory cytokines (e.g., TNF‐α, IL‐6), thereby improving histopathological architecture [8]. These findings correspond to the strong correlations observed between the AI, histological damage, and biochemical markers in our results. In agreement, it was emphasized the role of inflammatory cytokines in the pathogenesis of CIN, reinforcing the significance of inflammatory mediators such as TNF‐α in our experimental model [18]. Moreover, it was highlighted the importance of integrating functional biomarkers with histopathological evaluation, noting that tubular epithelial damage alone may not fully reflect renal dysfunction, an observation supported by our study's correlation between histopathological injury, apoptotic activity, and biochemical markers such as creatinine and urea levels [19]. Together, these studies support the multifactorial pathogenesis of CIN and underscore the renoprotective potential of riociguat as observed in our model.

The use of contrast agents in various imaging and interventional procedures has risen significantly due to technological advancements [20]. CIN is a complex syndrome of AKI that typically manifests 24–48 h after the administration of iodinated contrast media (CM) [12]. Recent years have seen an increase in CIN incidence, with associated cardiac and non‐cardiac events in affected patients [20, 21]. The pathogenesis of CIN, as elucidated through in vitro studies and experimental models, involves several mechanisms: (i) decreased renal blood flow due to renal artery vasoconstriction, leading to medullary hypoxia; (ii) direct tubular toxicity induced by CM, mediated through apoptosis and oxidative stress; (iii) endothelial dysfunction and alterations in renal microcirculation; (iv) increased plasma viscosity, resulting from CM‐induced resistance in the vasa recta, which further exacerbates medullary hypoxia and tubular ischemia. Understanding and identifying these underlying mechanisms is critical for developing strategies to prevent contrast nephropathy [22, 23]. Among these mechanisms, enhancing NO levels to alleviate renal artery vasoconstriction has been a focus of many studies aimed at reducing CIN [14, 24].

Riociguat is a drug approved by the World Health Organization for the treatment of pulmonary arterial hypertension, functioning as a stimulator of sGC. It operates on the NO‐guanylate cyclase‐cyclic guanosine monophosphate (cGMP) pathway by both directly stimulating guanylate cyclase and sensitizing sGC to NO, a potent vasodilator. When NO binds to guanylate cyclase in vascular smooth muscle cells, it increases cGMP production, which in turn induces vasodilation [25, 26]. It has been shown that CM reduces NO production, which can be counteracted by NO replacement, providing extracellular clearance and increasing regional blood flow to support medullary oxygenation. Previous studies have demonstrated the renoprotective effects of phosphodiesterase‐5 inhibitors, which increase cGMP levels, in preventing contrast nephropathy [25, 26, 27, 28].

Consistent with the literature, our study demonstrated decreased NO levels in the CIN group, while NO levels were found to increase in the riociguat treatment (CNM‐riociguat) group. Although riociguat does not directly induce NO release, this effect may be attributed to its ability to enhance receptor sensitivity, thereby triggering NO release in other cells [29]. sGC stimulators have been shown to exert various beneficial effects, including anti‐inflammatory, antiproliferative, and antifibrotic actions. Furthermore, sGC stimulation has been reported to reduce renal fibrosis and increase diuresis and natriuresis, demonstrating significant renoprotective effects [12, 30, 31]. In our study, histopathological examination of kidney tissue from the contrast nephropathy group revealed dilation in both distal and proximal tubules, vacuolization in tubular epithelial cells, severe desquamation, loss of the brush border, shrunken glomerular structure, Bowman's capsule degeneration, congestion, and edema. In contrast, these morphological damages were significantly alleviated in the riociguat + contrast nephropathy group. TUNEL staining to evaluate apoptotic cells revealed intense apoptosis in the proximal‐distal tubular epithelium of the contrast nephropathy group, while fewer apoptotic cells and more normal cells were observed in the riociguat + contrast nephropathy group. Tubular injury is a significant factor in renal tubular disorders, both acute and chronic, with glomerular damage serving as a key indicator of damage severity [2]. These findings suggest that riociguat may have a morphologically restorative effect on renal nephropathy by demonstrating antiapoptotic properties in CIN.

Among the detrimental effects of CM on renal tubular cells is the activation of proinflammatory cytokine pathways. TNF‐α is one of the primary proinflammatory cytokines responsible for tissue damage and inflammation [32]. In our study, TNF‐α levels were evaluated as a marker of the inflammatory response. We observed a significant increase in TNF‐α levels in the contrast nephropathy group, indicating an inflammatory response. However, a 1.7‐fold decrease in TNF‐α levels was noted in the riociguat + contrast nephropathy group compared to the CIN group. These findings suggest that riociguat reduces the proinflammatory response and exerts anti‐inflammatory effects.

Furthermore, the inclusion of NGAL and AOPP measurements provided additional mechanistic insight into the effects of riociguat. NGAL, a sensitive marker of tubular injury, was markedly elevated in the CIN group, reflecting significant tubular epithelial damage and stress. Treatment with riociguat led to a pronounced reduction in NGAL levels, indicating attenuation of tubular injury and preservation of epithelial integrity. Similarly, AOPP, as an indicator of oxidative protein damage, was significantly increased in CIN, corroborating the role of oxidative stress in CIN. Riociguat administration effectively decreased AOPP levels, demonstrating its capacity to mitigate oxidative stress and protect renal tissue from protein oxidation. These biochemical findings align with the histopathological and apoptotic observations, reinforcing the multifaceted protective effects of riociguat on renal structure and function.

However, several methodological considerations should be acknowledged. Serum NGAL was used as a supportive biomarker of tubular injury; nevertheless, NGAL levels may be influenced by systemic inflammatory processes and therefore do not represent a fully specific indicator of tubular necrosis. In the present study, histopathological scoring and TUNEL‐based AI were considered the primary measures of renal injury. In addition, sGC activity was measured only in serum, and renal tissue–level pharmacodynamic confirmation was not performed. Furthermore, the experimental design did not include separate comparator groups for sGC activators versus stimulators. Therefore, the mechanistic interpretation of riociguat's renoprotective effects should be considered preliminary.

Moreover, this study did not include proteinuria assessment, renal gene‐expression analyses, or protein‐level investigations of inflammatory mediators such as NF‐κB, IL‐1, IL‐6, and CCL2. TNF‐α was selected as a representative inflammatory marker, but the absence of a broader cytokine and molecular profile limits mechanistic interpretation. Future studies incorporating transcriptomic, proteomic, and immunohistochemical analyses are needed to better elucidate riociguat‐mediated pathways.

Importantly, fibrosis‐specific staining methods (e.g., Masson's trichrome or collagen immunostaining) were not performed because this study focused on an acute CIN model. Therefore, references to fibrosis have been revised, and the observed effects should be interpreted as improvements in acute structural injury rather than chronic fibrotic remodeling.

Finally, the ML analysis identified the AI as the dominant predictor of renal injury. Because this variable requires histological evaluation, the ML model should be interpreted as an exploratory mechanistic tool rather than a clinically applicable early‐detection or screening model. Future studies using non‐invasive biomarkers and external validation cohorts are required to establish clinical predictive utility.

In conclusion, treatment with riociguat attenuated histopathological damage, including tubular dilation, vacuolization, and apoptosis, suggesting its potential to preserve renal function and structure. Furthermore, riociguat exhibited anti‐inflammatory effects by significantly reducing TNF‐α levels, thereby suppressing the inflammatory response associated with CIN. Additionally, riociguat increased NO levels, which suggests its vasodilatory effects, contributing to the maintenance of renal perfusion and oxygenation.

The results of the feature importance and model performance analysis in this study, when considered alongside the findings from the referenced literature, highlight the consistency and effectiveness of ML applications in predicting health‐related outcomes. Specifically, the identification of AI and NO as key features, with AI contributing significantly to the model's predictive power, aligns with previous research [9], which emphasized the predictive strength of immune‐related biomarkers in disease outcomes. The successful application of ensemble methods like AdaBoost and CatBoost, as seen in this and other studies [10], underscores the importance of selecting biologically relevant features to enhance model accuracy. Furthermore, the integration of explainable AI (XAI) methods such as SHAP could provide deeper insights into the decision‐making processes of the model, as demonstrated by [33], improving interpretability and clinical relevance. Additionally, the robustness of the model's predictions, validated by studies such as [11], reinforces the critical role of apoptosis in understanding disease mechanisms. The clinical implications of these findings, particularly in the development of interpretable models for disease risk prediction, emphasize the value of ML not only in improving predictive accuracy but also in enhancing its practical applicability in clinical settings, as further exemplified by [34]. Integrating these approaches could significantly elevate the clinical relevance of this study, contributing to advancements in healthcare decision‐making.

These findings underscore the multifaceted therapeutic potential of riociguat in the prevention of CIN. By targeting intrarenal hemodynamic alterations and inflammatory pathways, riociguat represents a promising therapeutic strategy for managing CIN and preserving renal function. The observed protective effects of riociguat further highlight its potential clinical utility as a preventative treatment for CIN in high‐risk patient populations undergoing contrast‐enhanced procedures. However, additional prospective studies with larger sample sizes are essential to validate these findings and to better elucidate the underlying mechanisms of riociguat‐mediated renal protection. Furthermore, clinical trials are necessary to translate these preclinical observations into clinical practice and to assess the safety and efficacy of riociguat in preventing CIN in human subjects. Collectively, this study contributes to the expanding body of evidence supporting the therapeutic potential of riociguat in the management of CIN and emphasizes the need for continued exploration of novel pharmacological interventions to address this critical clinical challenge.

The literature reports conflicting results regarding the relationship between CIN and gender. Some studies suggest that the female gender may have a protective effect against the development of CIN, while others report no significant difference between genders [35, 36, 37]. In our study, only female rats were used, and therefore we do not make a direct comment on this issue. In addition, 12‐week‐old rats were selected for our study. Previous research has suggested that younger age may have a renoprotective effect [38]. The reason for selecting 12‐week‐old rats in our study was to create a relatively homogeneous group with a limited number of animals. A design including rats of both sexes and different age groups could have contributed to a more comprehensive evaluation of the relationship between CIN and gender or age.

### 4.1 Limitations

This study has several limitations. First, proteinuria was not measured, which limits the evaluation of renal functional outcomes. Additionally, due to resource and methodological constraints, renal gene expression and protein analyses could not be performed. Likewise, inflammatory markers other than TNF‐α—such as NF‐κB phosphorylation, CCL2, IL‐1, and IL‐6—were not assessed. The inclusion of gene expression data and additional inflammatory parameters would have provided more comprehensive mechanistic insights and allowed for a more detailed evaluation of renal injury and associated inflammatory pathways. Also, Although NGAL is an early indicator of tubular injury, serum levels may be affected by systemic inflammation, and histology or immunostaining is required to confirm true tubular necrosis.

Moreover, the study design did not include separate experimental groups for sGC activators and stimulators. Evaluating these agents individually could have contributed to a better understanding of their distinct mechanisms of action and added further mechanistic value to the study. In addition, sGC levels were measured only in serum samples, and assessing local effects in renal tissue could have provided additional insight.

Furthermore, this study was performed using a rat model, which may not fully capture the complex pathophysiology of CIN in humans. While the results demonstrate the protective effects of riociguat in this preclinical setting, caution is needed when extrapolating these findings to clinical practice. Additional limitations include the relatively small sample size and the absence of long‐term outcome assessments. Moreover, the detailed molecular mechanisms underlying riociguat's renoprotective effects require further investigation. Future studies with larger animal cohorts, extended follow‐up periods, and clinical trials are necessary to confirm and translate these findings to human patients.

## 5 Conclusion

This experimental study demonstrates the therapeutic potential of riociguat in preventing CIN. Riociguat treatment significantly reduced contrast medium‐induced renal injury in rats, improving histopathological findings such as tubular damage and inflammation (without evidence of fibrosis‐specific assessment, as no dedicated staining or quantitative collagen analysis was performed), and lowering SCr and blood urea nitrogen levels. Additionally, riociguat reduced oxidative stress and tubular injury, as indicated by lower AOPP and NGAL levels.

Correlation analysis revealed strong associations between structural damage, apoptosis, and biochemical markers, with TNF‐α linked to apoptotic activity. Machine learning models, particularly XGBoost, identified AI as the most influential predictor, followed by NO, highlighting the roles of apoptosis and oxidative stress in CIN pathophysiology. Because AI is derived from histopathological evaluation, these findings primarily provide mechanistic insight rather than supporting claims of early clinical detection or screening utility.

These findings suggest riociguat as a promising candidate for CIN prevention. Future studies should include fibrosis‐specific histological techniques (e.g., Masson's trichrome staining or collagen marker analysis) and external validation cohorts to confirm clinical applicability and long‐term outcomes.

## Funding

The support was received as a project from Harran University—Scientific Research Projects unit for this study (number: 22169).

## Ethics Statement

The study was conducted with the approval of the Harran University Animal Experiments Local Ethics Committee (HADYEK) (Study protocol license no. 2022/002/12).

## Conflicts of Interest

The authors declare no conflicts of interest.

## Data Availability

The data that support the findings of this study are available on request from the corresponding author. The data are not publicly available due to privacy or ethical restrictions.
